# Reply: Childhood leukaemia incidence and the population-mixing hypothesis in US SEER data

**DOI:** 10.1038/sj.bjc.6602433

**Published:** 2005-03-01

**Authors:** D Wartenberg

**Affiliations:** 1 Division of Environmental Epidemiology, Department of Environmental and Occupational Medicine, UMDNJ – Robert Wood Johnson Medical School, 170 Frelinghuysen Road, Piscataway, NJ 08854, USA

**Sir**,

We thank Parslow *et al* for their interest in our paper (Wartenberg *et al*, 2004) and apologise for any lack of clarity on our part. In listing 16 previous studies on population mixing and childhood leukaemia, we did not state that they all supported Kinlen's hypothesis, but that they were ‘similar investigations…and obtained *largely* similar results.’ Thus, they all addressed the aetiology of this disease using standard epidemiologic designs (i.e., 10 ecologic, three cohort, two space–time clustering, one case control), and most (12 of the 16) reported evidence supporting the rural–urban population-mixing hypothesis (Kinlen, 1988). The study by Parslow *et al* (2002) that prompted their letter is one of four that did not show any excess of childhood leukaemia, but then this is not surprising since, like that by Law *et al* (2003), it concerned, not rural, but largely urban population mixing (of undetermined onset) in which an excess would not be expected on the Kinlen hypothesis.

Most of their letter criticises previous work mainly by Kinlen, characterising it as ‘selecting rural areas with sudden increases of population mixing’ and involving ‘relatively small populations’. In fact, our own study involved large populations chosen independently of socio-demographic events, while other studies, far from only examining areas of marked rural population mixing, were national in scope, including wartime evacuation of children (Kinlen and John, 1994), national servicemen (Kinlen and Hudson, 1991) and the North Sea oil industry (Kinlen *et al*, 1993).

It is unusual to concentrate first on protective or immune effects in studying infectivity in preference to what produces an excess of the disease in question. The study of rural–urban population mixing focuses on situations conducive to what is central, namely the transmission of any underlying infection in childhood leukaemia from infected to susceptible individuals, the latter being more prevalent in rural areas. The results of this approach have been most encouraging in finding excesses of the disease (Kinlen and Doll, 2004; Wartenberg *et al*, 2004). Thus, our study showed that the population-mixing effect was most prominent in rural counties that also were isolated.

An alternative explanation for the protective effect of high levels of *urban* population mixing reported by Parslow *et al* is decreased susceptibility of the children in such areas as a result of the greater herd immunity that tends to typify urban areas. Consistent with this is the reduced incidence associated with population mixing in our study when urban and rural areas were combined (Wartenberg *et al*, 2004), as well as in urban areas in other studies (Kinlen and Hudson, 1991; Koushik *et al*, 2001).

To regard the various studied examples of rural population mixing as unsatisfactory because they involve different definitions is hardly reasonable. What is relevant is that all (including our own) were prompted by the same basic hypothesis; people come together in a variety of different settings and, accordingly, so do outbreaks occur of infection-based illnesses. Parslow *et al*'s own definition of population mixing is one of many surrogates that have been used, each with their own strengths and limitations, including measures of changes in population size over time, population density, maternal infection during pregnancy, day-care attendance, vaccination, early childhood infectious exposures, migration patterns and even space–time clustering. Each of these may help us better understand the infectious aetiology of childhood leukaemia, if results are assessed, compared and integrated carefully, rather than discarded, because they do not fit a preconceived notion of the optimal measure. One limitation of Parslow *et al*'s index is its dependence upon a single census source, inextricably combining recent and long-standing (urban) mixing, each of unknown degree. While the choice of measure used is often dictated by the type of data available, where possible it would be valuable to compare results within and across data sets using multiple measures, in part, to note their lack of independence, and in part, as a type of sensitivity analysis.
